# Physical and Social Environment Are Associated to Leisure Time Physical Activity in Adults of a Brazilian City: A Cross-Sectional Study

**DOI:** 10.1371/journal.pone.0150017

**Published:** 2016-02-25

**Authors:** Crizian Saar Gomes, Fernanda Penido Matozinhos, Larissa Loures Mendes, Milene Cristine Pessoa, Gustavo Velasquez-Melendez

**Affiliations:** 1 Universidade Federal de Minas Gerais, Department of Maternal and Child Nursing and Public Health, Av. Alfredo Balena, 190, Santa Efigênia, 30130–100 Belo Horizonte, Minas Gerais, Brazil; 2 Universidade Federal de Minas Gerais, Department of Nutrition, Av. Alfredo Balena, 190, Santa Efigênia, 30130–100 Belo Horizonte, Minas Gerais, Brazil; 3 Universidade Federal de Ouro Preto, Department of Clinical and Social Nutrition, R. Diogo de Vasconcelos, 122, Pilar, 35400–000, Ouro Preto, Minas Gerais, Brazil; Vanderbilt University, UNITED STATES

## Abstract

The physical activity practice is highlighted as a strategy to health promotion and to avoid chronic diseases. In addition to individual factors, environmental characteristics in which people live, may offer opportunities or barriers in adopting healthy habits and this is related to the physical activity (PA) practice among individuals. The aim of this study is to investigate the associations between neighborhood environment and leisure-time physical activity in adults. This is a cross-sectional study, developed using the database of Surveillance System for Risk and Protective Factors for Chronic Diseases by Telephone Survey (VIGITEL 2008/2010) of Belo Horizonte, Brazil. Individuals with the habit of practicing PA for at least 150 minutes of moderate-intensity PA or at least 75 minutes of vigorous-intensity PA throughout the week in leisure time were classified as active in leisure time. To characterize the built and social environment we used georeferenced data of public and private places for physical activity, population density, residential density, homicide rate and total income of the coverage area of the basic health units. The covered area of the basic health units was used as context unit. For data analysis, we used multilevel logistic regression. The study included 5779 adults, 58.77% female. There was variability of physical activity in leisure time between area covered by the basic health units (Median Odds ratio = 1.30). After adjusting for individual characteristics, the increase of density of private places for physical activity (Odds ratios—OR = 1.31; 95% confidence interval—95% CI: 1.15 to 1.48) and the smaller homicide rate (OR = 0.82; IC95%: 0.70 to 0.96) in the neighborhood increased physical activity in leisure time. The evidence of this study shows that neighborhood environment may influence the physical activity practice in leisure time and should be considered in future interventions and health promotion strategies.

## Introduction

Regular physical activity (PA) is essential for disease prevention, particularly non-communicable chronic diseases (NCDs); health promotion; quality of life; and reduced mortality[1– 2].

According to 2010 Global Burden of Disease (GBD 2010) study data, physical inactivity and insufficient PA accounted for approximately 3.2 million deaths (range, 2.7–3.7 million) and 2.8% (range, 2.4%–3.2%) of disability-Adjusted Life Year (DALY)[3].

The beneficial effects of PA are well documented in the scientific literature; however, the prevalence of activity among individuals remains low, especially during leisure time, in both developed and developing countries[4].

For decades, research has focused on assessing individual characteristics as determinants of PA. Interventions addressing individual factors explain much of PA practice, but are insufficient to increase PA at population levels. Thus, increasing attention has been paid to ecological PA models, in which the assumption is that the environments in which people live are important for promoting healthy habits, as they may offer opportunities or barriers to PA, and also that this influences can be different for each PA domains [5].

In general, studies show that increased availability of green areas and places for PA, street connectivity, favorable perceptions of neighborhood (pleasant aesthetics, traffic and crime safety), higher population and residential density and more mixed land use are associated with increased levels of leisure time physical activity (LTPA) [6–9].

Studies in developed countries provide the most consistent evidence for the role of environment features in LTPA. However, these findings may not apply to population in low-and-middle income countries such as Brazil, since it is observed differences in the historic process of urban center formation and recent changes in social and physical environments, as well as those related to urban planning [9,10].

Studies about this topic in Brazil have focused on individual perceptions of the environment, and there is limited evidence on the association between PA and neighborhood physical and social environments based on geo-referenced data [7,9, 11–13]. Most studies have not conducted with a representative sample or consider only defined city areas. Many studies also did not consider the hierarchical structure of data and not adjust for potential confounding variables in their analysis, which can lead to overestimation of the effects.

Urban contextual characteristics of Brazilian cities based on geographic information system (GIS) and data from NCD surveillance system surveys conducted since 2006 can provide data to evaluate the relationship between exposures to neighborhood physical and social environments and PA levels.

To strengthen existing evidence on the role of neighborhoods on PA, we investigated associations between neighborhood environment and LTPA in adults.

## Methods

This cross-sectional study included adults who participated of the Surveillance System for Risk and Protective Factors for Chronic Diseases (Vigitel), in the years 2008, 2009, and 2010 in the city of Belo Horizonte, the state capital of Minas Gerais. This city is located in southeastern Brazil and covers a total area of 331 km^2^; the city has a population of 2,365,151 inhabitants and a population density of 7,177 inhabitants/km^2^ [14].

Vigitel survey is accomplished annually by telephone interview and evaluates risk and protective factors for non-transmittable chronic diseases among adults living in the all capitals of Brazilian states, includes demographic characteristics and health behavior and questions regarding physical activity. Detailed information about the Vigitel has been described elsewhere [15].

In 2008, 2009, 2010, a total of n = 6,034 individuals adults (≥18 years) were assessed. After excluding pregnant women (n = 43), women who did not know they were pregnant at the time of the interview (n = 4), and individuals who did not have quality address information (n = 175 with a blank postal code, one with a postal code from another municipality, and 32 invalid postal codes), the final sample consisted of 5,779 adults.

LTPA was assessed based on responses to the questions: "In the last three months, have you practiced some kind of physical exercise or sport?; "What is the main type of exercise or sport you have practiced?"; "Do you exercise at least once a week?"; "How many days per week do you practice physical activity or exercise?"; "On the days that you exercise or participate in sports, how long does this activity last?". We considered as active during leisure time the individuals that practice at least 150 minutes of moderate PA per week or 75 minutes of vigorous PA per week [16].

Urban areas named as Coverage Areas of Basic Health Units (CABHUs) were used as the neighborhood unit in this study. CABHUs are a set of census tracts units territorially delimited according to administrative and sanitary criteria, with average area of 2.55Km^2^ (±1,74Km^2^) each and average population of 20811 (±10541) inhabitants. The CABHUs was used since it is the administrative unit used to organize the health action and public policies including basic health care services and also by the fact that census tracts are very small areas where there would be a higher possibility of the studied variables homogeneity. The city of Belo Horizonte is divided into 148 CABHUs.

The CABHU that each participant belongs was determined based on geographical coordinates (latitude and longitude) obtained from residential postal codes (CEPs). Georeferencing of contextual variables was based on the address of the location obtained from commercial and government sources.

The exposure variables in this study were based on literature review and selected from the VIGITEL database and available georeferenced data.

The contextual characteristics evaluated in this study included:

- density of public places for PA (obtained in 2012): number of parks, public squares and lanes, and Academia da Saúde (Health Academy) in the CABHUs/area (km^2^) CABHUs. Health Academy is a program by the Brazilian Ministry of Health, which aims to offer physical activity classes in community settings at no cost to participants in Brazilian municipalities[17];
- density of private places for PA (obtained in 2011): number of sport and dance schools, gyms, sporting, and social clubs in the CABHUs/ area (km^2^) CABHUs;
- population density (obtained in 2010): population of CABHUs/ area (km^2^) CABHUs;
- homicide rate (obtained in 2009): number of homicides of CABHUs / population of CABHUs;
- residential density (obtained in 2010): number of households CABHUs/ area (Km^2^) CABHUs;
- total CABHUs income (obtained in 2010):Total nominal monthly income of individuals 10 years old or more of CABHUs.

Individual variables measured in this study included education, gender, age, marital status, skin color, consumption recommended of fruits or vegetables (FV) (five or more per day for five or more days of the week), consumption of meat with visible fat, consumption of sweetened beverages five or more days per week, smoking, and perceived poor health status.

Multilevel logistic regression of fixed effects with random intercepts was used because the data had variables related to both individuals (level 1) and to CABHU (level 2). Modeling was performed in three stages: Model 1 included only the random intercept to detect contextual effects; Model 2 included only individual variables; and Model 3 included both individual and contextual variables.

Fixed effects were presented as odds ratios (OR) with 95% confidence interval (95% CI); random effects were presented as CABHUs variance and standard error (SE).The variance was also translated to median odd ratios (MOR). MOR quantifies, in OR scales, LTPA variation between CABHUs (second-level variation). MOR values are always greater than 1; values equal to 1 mean that there is no variation between CABHUs [18,19].

To quantify the contribution of contextual variables to the overall between CABHUs variation in PA, we used the 80% interval of odds ratios (IOR 80%). The IOR is a measure of fixed effects that provides a interval for the odds ratio; it includes 80% of values of two randomly chosen individuals with the same individual characteristics and different measures of neighborhood covariate. So it incorporates both the fixed contextual variables effect and the unexplained between CABHUs heterogeneity. If interval count value of 1 indicates residual variability, in other words, the contextual variables alone cannot explain variability between CABHUs. The IOR therefore complements the information provided by the conventional OR [18,19].

The model adjustment was assessed using Akaike Information Criterion (AIC); the best model was the one with the smallest AIC [18,20].

All analyses were performed using Stata 12.1, and it were considered individual weightings based on the inverse of the number of telephone lines and number of adults in the interviewed household. A 5% significance level was considered for analysis of the final model. As the Vigitel is a telephone survey, written informed consent was replaced by verbal consent obtained from the respondent at the time of telephone contact. All interviews were recorded. This study was approved by the Ethics Committee of the Ministry of Health of Brazil, which waived the need for written consent and, Research Ethics Committee of the Federal University of Minas Gerais (ruling No. 25447414.1.0000.5149).

## Results

We analyzed 5,779 participants, residents in 148 CABHUs; among them, 58.77% were female and 39.29% had 9–11 years of education. Most participants were skin color mulatto/brown (51.79%) and lived with a partner (47.17%). The average age was 44 years (SD = 17) (Table 1).

**Table 1:** Characteristics of study participants. Belo Horizonte, Brazil. VIGITEL, 2008–2010. Notes: SE: standard error

Characteristics	% (SE)
**Gender**	
Male	41.23 (0.72)
Female	58.77 (0.72)
Age (years)*	44.00 (17.00)
**Skin color**	
White	40.36 (0.71)
Black	7.45 (0.39)
Mulatto/Brown	51.79 (0.73)
Others	0.40 (0.08)
**Education (years of study)**	
0 to 8	33.07 (0.68)
9 to11	39.29 (0.71)
12 or more	27.64 (0.65)
**Marital Status**	
Single	39.28 (0.74)
Married	47.17 (0.74)
Widow/Separated/Divorced	13.55 (0.47)
**Leisure time Physical activity**	
Yes	34.25 (0.69)
No	65.75 (0.69)

* mean and standard deviation

Only 34.25% of subjects engage in LTPA (Table 1). Among 148 CABHUs, the prevalence of LTPA varied from 0 to 75% (95% CI = 5.79 to 97.96). There was an average of 39 individuals in each CABHUs, ranging from 2 to 167.

Unadjusted analysis (Table 2) indicated that male gender, higher education, consuming the recommended of FV, not consuming of meat with visible fat, not smoking, not have a poor self-perceived health status, highest density of private places for PA practice, and higher relative income per CABHUs were associated with increased odds of LTPA. In contrast increasing age, married and widowed/separated/divorced, and higher homicide rates in CABHUs were associated with lower odds of LTPA.

**Table 2:** Unadjusted analysis of factors associated with being sufficiently activity in leisure time. Belo Horizonte, Brazil. VIGITEL, 2008–2010. Notes: SE: standard error; OR: odds ratio; 95% CI: 95% confidence interval; FV: fruits and vegetables; PA: physical activity.

Factors	Yes	No	OR (95% CI)
	% (SE)	% (SE)	
**Individual-level**			
**Gender**			
Female	28.41 (0.85)	71.59 (0.85)	**-**
Male	42.59 (1.13)	57.41 (1.13)	1.89 (1.66–2.14)
Age*(years)	41.51 (0.42)	45.24 (0.29)	0.98 (0.98–0.98)
**Skin color**			
White	36.48 (1.09)	63.52 (1.09)	-
Mulatto/brown	32.88 (0.96)	67.12 (0.96)	0.89 (0.68–1.15)
Black	32.42 (2.60)	67.58 (2.60)	0.89 (0.77–1.03)
Others (red/yellow)	20.51 (8.86)	79.49 (8.86)	0.50 (0.17–1.47)
**Education (years)**			
0 to 8	23.08 (1.05)	76.92 (1.05)	-
9 to 11	36.74 (1.14)	63.26 (1.14)	1.93 (1.63–2.28)
12 or more	44.07 (1.36)	55.93 (1.36)	2.59 (2.19–3.06)
**Marital Status**			
Single	39.46 (1.23)	60.54 (1.23)	-
Married	32.38 (0.99)	67.62 (0.99)	0.74 (0.65–0.85)
Widow/Separate/Divorced	28.20 (1.65)	71.80 (1.65)	0.59 (0.49–0.71)
**Consumption of FV**			
No	30.15 (0.79)	69.85 (0.79)	-
Yes	45.51 (1.38)	54.49 (1.38)	1.90 (1.67–2.16)
**Consumption of sweetened beverages**			
No	34.73 (0.78)	65.27 (0.78)	-
Yes	32.54 (1.50)	67.46 (1.50)	0.93 (0.79–1.10)
**Consumption of meat with visible fat**			
Yes	31.83 (1.15)	68.17 (1.15)	-
No	35.64 (0.87)	64.36 (0.87)	1.15 (1.00–1.31)
**Smoker**			
Yes	27.89 (1.76)	72.11 (1.76)	-
No	35.28 (0.75)	64.72 (0.75)	1.46 (1.19–1.78)
**Perception of poor health status**			
Yes	11.60 (2.50)	88.40 (2.50)	-
No	35.04 (0.71)	64.96 (0.71)	3.88 (2.45–6.13)
** ****Contextual-level**	**Mean (SE)**	**Mean (SE)**	**OR (95% CI)**
Density of public places for PA practice (number/km^2^)	0.58 (0.01)	0.57 (0.00)	1.48‡ (0.49–4.48)
Density of private places for PA practice (number/km^2^)	4.92 (0.11)	3.96 (0.07)	1.54‡ (1.32–1.80)
Population density (inhabitants/km^2^)	9704.59 (83.50)	9716.29 (60.41)	1.02^┼^ (0.83–1.26)
Homicide rate (per 10,000 inhabitants)	5.64 (0.08)	6.11 (0.06)	0.71‡ (0.60–0.83)
Total income	4027.00 (105.94)	3138.75 (64.09)	1.70^╫^ (1.39–2.09)
Residential density (house/km^2^)	3199.30 (29.93)	3141.06 (20.00)	1.00‡ (0.99–1.01)

*Mean and standard deviation

‡ Results for increment of 10 units

^**┼**^ Results for increment of 10,000 inhabitants/km^2^

^╫^Results for increment of R$ 10,000.00.

Table 3 shows ORs and 95% CI of individual and contextual characteristics for the three multilevel logistic regression models used in this study.

**Table 3:** Multilevel logistic regression models for being sufficiently activity in leisure time. Belo Horizonte, Brazil. VIGITEL, 2008–2010. Notes: PA = physical activity; OR: odds ratio; 95% CI: 95% confidence interval; SE: standard error; AIC: Akaike information criterion; Model 1: empty model; Model 2: model with individual variables; Model 3: model with contextual variables;

Variables	Model 1	Model 2	Model 3
**Fixed effects**	**OR (95% CI)**	**OR (95% CI)**	**OR (95% CI)**
Age (years)		0.99 (0.98–0.99)	0.99 (0.98–0.99)
Gender (male/female)		2.07 (1.81–2.37)	2.07 (1.81–2.37)
Education (years)			
9 to 11 vs. 0 to 8		1.57 (1.31–1.87)	1.50 (1.25–1.79)
>12 vs. 0 to 8		1.93 (1.62–2.29)	1.74 (1.47–2.06)
Consumption of fruits and vegetables (yes/no)		1.98 (1.73–2.27)	1.98 (1.73–2.27)
Consumption of meat with visible fat (no/yes)		1.24 (1.08–1.42)	1.22 (1.06–1.40)
Perception of poor health (no/yes)		2.70 (1.69–4.32)	2.68 (1.68–4.26)
Current smoker (no/yes)		1.34 (1.09–1.65)	1.36 (1.10–1.67)
Density of private places for PA (number/km^2^)			1.31‡ (1.15–1.48)
80% interval of odds ratio			0.96–1.10
Homicide rate (per 10,000 inhabitants)			0.82‡ (0.70–0.96)
80% interval of odds ratio			0.92–1.05
**Random effect**			
**Coverage Areas of Basic Health Units**			
Variance (SE)	0.07 (0.02)	0.03 (0.02)	0.00 (0.01)
Reduction of variance (%)		59.10	95.60
Median OR (MOR)	1.30	1.18	1.03
AIC	7413.38	6962.94	6944.05

‡ Results for increments of 10 units.

The null model (Model 1) indicates existence of LTPA variability between CABHUs (MOR = 1.30). After including individual and contextual variables (Models 2 and 3), we observed reduced MOR (MOR = 1.18 and MOR = 1.03, respectively), suggesting that these variables contribute to the LTPA variability between CABHUs. Individual characteristics explained 59.10% of the variance of level 2. After adjusting for individual variables, contextual variables explained 95.60% of the variance; however, the MOR of 1.03 indicates that a small amount of variation remains unexplained.

After adjusting for individual variables, the density of private places were positively associated (OR = 1.31; 95% CI: 1.15 to 1.48) and the homicide rate (OR = 0.82; 95% CI: 0.70 to 0.96) negatively associated to likelihood of LTPA (Model 3). The IORs of places to practice PA and homicide rate was 0.96–1.10 and 0.92–1.05, respectively. Both intervals contain the value 1, which suggests that these variables do not fully explain heterogeneity between CABHUs.

In assessing model adjustment, we observed reduced AIC values after including individual and contextual-level variables (Table 3).

## Discussion

The findings of this study suggest that the environments in which people live have characteristics that may affect LTPA. In this study, individuals residing in CABHUs with greater availability of private facilities for PA and lower crime rates have highest odds to practice LTPA. This study offers new perspectives for understanding the relationship between urban built environments and PA using local or national surveillance systems.

The positive association between the presence of private exercise facilities and LTPA in this study is consistent with reported findings in high-income [21–23], and Latin America countries [7,24]. Current evidence indicates that people are more active during leisure time when they have access to facilities PA, since the availability of places to exercise near the place of residence draws attention and serves as visual stimuli to encourage PA [7,22]. Furthermore, the main reason for not performing or to stop practicing PA is the lack of facilities or geographic proximity. Thus, physical proximity can reduce psychological and physical barriers associated with exercise [25,26]. A study in a Brazilian city reported that people living in areas with the highest number of gyms (≥2) had increased chances of meeting current PA recommendations [7].

Unlike the association between private resources and LTPA, the presence of public resources was not associated with LTPA. Similar observations were reported in Curitiba (Brazil) [7] and San Diego (United States) [22]. However, other studies, both national and international, reported a positive association between the availability of public places and LTPA [27–31]. The small number of public resources for practice PA in Belo Horizonte may have been insufficient to detect an association. Moreover, it is unclear whether these public places are safe and comfortable for use.

Safe community was also a factor associated with LTPA, with higher homicide rates in CABHUs associated with reduced LTPA. These findings are particularly relevant in low- and middle-income countries, where insecurity and crime are increasing due to rapid urbanization [32]; and in adults, because crime is markedly higher overnight and most people work during the day, they can only perform LTPA at night. Insecurity breeds fear, which can limit mobility and diminish confidence in traveling by foot or practicing PA outdoors, thus contributing to a reduced PA [33,34].

The association between community safety and LTPA is not consistently reported in the literature [34–36]. Different safety indicators in these studies may partially explain the controversy over the role of urban crime and PA. Most studies evaluate safety subjectively (based on participant perception) [37]. These perceptions may not reflect reality because individuals may exaggerate security problems. For this reason, official city statistics generated by local agencies can better reflect crime rates.

A study conducted in southern Brazil showed that the perception of crime and feelings of security were positively associated with LTPA [38]. People who perceived their neighborhood safety to be excellent were 35% more likely to be active during leisure time compared to those who believed their neighborhoods were unsafe [39]. Amorim *et al*. [40] and Weber-Corseuil *et al*. [41] reported similar observations in different regions of Brazil. However, using similar instruments, Gomes *et al*. [42], Hallal *et al*. [43], Mendes *et al*. [44], Parra *et al*. [45], and Rech *et al*. [46] found no association between security and LTPA.

The current study is one a few to assess the relationship between environment and PA in Latin America countries using objective contextual measures. The strengths of this study included the sample representativeness of a Brazilian city, the high rate of georeferencing (96.55%), adjustment for individual variables, and use of multilevel analytical methodologies.

This study had several limitations. Its cross-sectional design limits temporal inferences. PA was evaluated based on information obtained by telephone surveys, which may be less accurate estimates of PA levels. However, validation studies of PA indicators showed satisfactory results and performed well in sensitivity and specificity analyses [47, 48]. The fact that these results were similar to those reported by the majority of other national and international studies argues in favor of the validity of these findings. Another limitation is the fact that the sample studied was composed of people living in households with fixed phone lines. However, landline coverage in Belo Horizonte is higher than the national average, which reduces the potential for selection bias [49]. It is recognized as an additional limitation to the use of CABHUs as neighborhood unit. The use of geographical or administrative units are often used as neighborhood boundaries, but may be inconsistent with what individuals consider / realize how neighborhood.

## Conclusions

The findings of this study support the hypothesis that social and physical barriers may partially explain variability in LTPA. Promotion of PA is a public health priority that requires effective intervention strategies. This study adds to current evidence and has implications for public health interventions aimed at increasing PA. Of note is that increased availability of leisure facilities and improved safety may be important investments for increased LTPA levels.
